# The Use of Oral Anticoagulation Is Not Associated With a Reduced Risk of Mortality in Patients With COVID-19: A Systematic Review and Meta-Analysis of Cohort Studies

**DOI:** 10.3389/fphar.2022.781192

**Published:** 2022-03-31

**Authors:** Meng-Fei Dai, Si-Tong Guo, Yi-Jun Ke, Bao-Yan Wang, Feng Yu, Hang Xu, Zhi-Chun Gu, Wei-Hong Ge

**Affiliations:** ^1^ Department of Pharmacy, China Pharmaceutical University Nanjing Drum Tower Hospital, Nanjing, China; ^2^ School of Basic Medicine and Clinical Pharmacy, China Pharmaceutical University, Nanjing, China; ^3^ Department of Pharmacy, The People’s Hospital of Guangxi Zhuang Autonomous Region, Nanning, China; ^4^ Department of Pharmacy, The Anqing Hospital Affiliated to Anhui Medical University, Anqing, China; ^5^ Department of Pharmacy, Nanjing Drum Tower Hospital, The Affiliated Hospital of Nanjing University Medical School, Nanjing, China; ^6^ Department of Pharmacy, Renji Hospital, Shanghai Jiao Tong University School of Medicine, Shanghai, China

**Keywords:** COVID-19, oral anticoagulant, direct oral anticoagulants, vitamin K antagonists, mortality, meta-analysis

## Abstract

**Background:** Hypercoagulability and thromboembolic events are associated with poor prognosis in coronavirus disease 2019 (COVID-19) patients. Whether chronic oral anticoagulation (OAC) improve the prognosis is yet controversial. The present study aimed to investigate the association between the chronic OAC and clinical outcomes in COVID-19 patients.

**Methods:** PubMed, Embase, Web of Science, and the Cochrane Library were comprehensively searched to identify studies that evaluated OAC for COVID-19 until 24 July 2021. Random-effects model meta-analyses were performed to pool the relative risk (RR) and 95% confidence interval (CI) of all-cause mortality and intensive care unit (ICU) admission as primary and secondary outcomes, respectively. According to the type of oral anticoagulants [direct oral anticoagulants (DOACs) or vitamin K antagonists (VKAs)], subgroup and interaction analyses were performed to compare DOACs and VKAs. Meta-regression was performed to explore the potential confounders on all-cause mortality.

**Results:** A total of 12 studies involving 30,646 patients met the inclusion criteria. The results confirmed that chronic OAC did not reduce the risk of all-cause mortality (RR: 0.92; 95% CI 0.82–1.03; *p* = 0.165) or ICU admission (RR: 0.65; 95% CI 0.40–1.04; *p* = 0.073) in patients with COVID-19 compared to those without OAC. The chronic use of DOACs did not reduce the risk of all-cause mortality compared to VKAs (*P*_interaction_ = 0.497) in subgroup and interaction analyses. The meta-regression failed to detect any potential confounding on all-cause mortality.

**Conclusion:** COVID-19 patients with chronic OAC were not associated with a lower risk of all-cause mortality and ICU admission compared to those without OAC, and the results were consistent across DOACs and VKA subgroups.

**Systematic Review Registration:** clinicaltrials.gov, identifier CRD42021269764.

## 1 Introduction

Coronavirus disease 2019 (COVID-19) is a respiratory infection caused by severe acute respiratory syndrome coronavirus-2 (SARS-CoV-2) with a high rate of morbidity and mortality (Wu and McGoogan, 2020). It has been recognized as a pandemic by the World Health Organization (World Health Organization, 2021). As of February 2022, >400 million patients have been confirmed with COVID-19 globally, and >5 million people have deceased (https://coronavirus.jhu.edu/map.html) (John Hopkins University, 2022). Although COVID-19 primarily affects the respiratory system, SARS-CoV-2 infection can lead to vascular inflammation and endothelial dysfunction due to hypoxia and excessive inflammation. A consequent hypercoagulability causes pulmonary embolism with micro- and macro-thrombosis in the lung vessels (Ackermann et al., 2020; Belen-Apak and Sarıalioğlu, 2020; Varga et al., 2020), rendering it as one of the main reasons for death and poor prognosis in COVID-19 patients. Previous studies have shown that venous thrombosis is common in patients infected with SARS-CoV-2, with an estimated venous thromboembolism (VTE) incidence of 25% (Cui et al., 2020; Zhang et al., 2020). Also, thrombosis was observed in autopsy studies (Wichmann et al., 2020).

The guidelines recommend that prophylactic low-molecular weight heparin (LMWH) should be administrated to all hospitalized patients with COVID-19 to decrease mortality (Moores et al., 2020). Nonetheless, according to recent studies, despite the use of apparently adequate thrombosis prophylaxis, COVID-19 patients have a higher incidence of VTE than those with other infectious diseases (Cui et al., 2020; Zhang et al., 2020; Spiegelenberg et al., 2021). Approximately half of VTE incidence occurred at or within 24 h of admission (Lodigiani et al., 2020). Therefore, it was hypothesized that in the early stages of COVID-19, the transition from asymptomatic to severe respiratory failure might be sudden, and the anticoagulation therapy started after the advanced stage of COVID-19 may be insufficient (Aslan et al., 2021). The oral anticoagulation (OAC) treatment prior to the earliest stages of COVID-19 infection may exert a protective effect for COVID-19-related outcomes.

Several studies have evaluated the effects of chronic therapeutic OACs in reducing the risk of mortality in patients with COVID-19; however, the conclusions were controversial (Aslan et al., 2021; Buenen et al., 2021; Gülcü et al., 2021; Spiegelenberg et al., 2021), and high-quality evidence is limited. Hitherto, no systematic review and meta-analysis have been conducted to investigate the association between chronic OAC and clinical outcomes in COVID-19 patients. Furthermore, the protective effects of the different types of oral anticoagulants, direct oral anticoagulants (DOACs), or vitamin K antagonists (VKAs) in COVID-19 patients are controversial. Therefore, this systematic review and meta-analysis were conducted to investigate the benefits and hazards of chronic OACs in COVID-19 patients comprehensively and rigorously.

## 2 Materials and Methods

The present study was performed in accordance with the Preferred Reporting Items for Systematic Reviews and Meta-Analyses (PRISMA) statement. The protocol for this review was prospectively registered in PROSPERO (CRD42021269764).

### 2.1 Data Sources and Search Strategy

The following electronic databases were searched comprehensively to identify all the potentially eligible articles from inception to 24 July 2021, without language restriction: PubMed, Embase, Web of Science, and Cochrane Library. In brief, the search strategy was identified based on the PICOS format, including three key concepts: 1) COVID-19, 2) oral anticoagulant, and 3) human study (Supplementary Table S1). The detailed search strategy is presented in Supplementary Table S2. For each of the concepts, the authors mapped the relevant keywords and Medical Subject Headings (MeSH). Also, the references were assessed manually to ensure that all the relevant studies were included. Two authors (MFD and STG) independently searched the databases, and any disagreements were resolved by consulting a third author (HX).

### 2.2 Study Selection and Outcomes

Studies were considered eligible and included if they 1) were randomized controlled trials (RCTs) or cohort studies; 2) consisted of patients with confirmed COVID-19; 3) included patients in the exposure group with chronic OAC; and 4) with outcomes including mortality or intensive care unit (ICU) admission. The exclusion criteria were as follows: 1) studies with lack of a comparison group; 2) review articles, case reports, case–control studies, cross-sectional studies, and clinical trial registries; and 3) studies with unrelated outcomes or unreported outcomes. All medical interventions after hospital admission, including anticoagulation and pharmacological treatments for COVID-19, were performed at the discretion of the medical team. The primary outcome was all-cause mortality, defined as death from any cause in COVID-19 patients. The secondary outcome was ICU admission.

### 2.3 Data Extraction

Information was extracted using a pre-specified form consisting of items, such as the first author’s name, publication year, study design, country, patient demographics, clinical characteristics, sample size, the type of the anticoagulant, and outcomes.

The adjusted effect estimates were preferred to the unadjusted effect estimates and were extracted for reducing the confounding factors. Also, the effect estimates and tabular data obtained from studies comprising propensity-matched groups were treated as adjusted estimates. If the reports did not provide adjusted effect estimates, we extracted crude effect estimates. The data were extracted by two authors (MFD and STG) independently and cross-checked by a third author (HX) for completeness and accuracy.

### 2.4 Quality Assessment

Two authors (MFD and STG) independently assessed the methodological quality of each study. The Cochrane Collaboration Risk of Bias Tool (Higgins et al., 2011) was used to identify the risk of bias of RCTs. The Newcastle–Ottawa Scale (NOS) score was determined to be nine while assessing three quality domains, selection, comparability, and outcome in the cohort studies (Wells et al., 2021). The total NOS score <5 points defined a study with a potentially high risk of bias (Wells et al., 2021). An additional reviewer (HX) was consulted to resolve any discrepancies.

### 2.5 Statistical Analysis

Relative risk (RR) and 95% confidence interval (CI) were calculated for the predetermined outcomes and pooled in the random-effects model due to the heterogeneity of study populations. The generic inverse variance method was used for weighing (Cumpston et al., 2019). The hazard ratio (HR) was also calculated when available. The rare case hypothesis was not used when the outcome incidence was >20%, and the odds ratio (OR) and HR were transformed to RR based on the approximation suggested by VanderWeele (2020). *I* ^*2*^ was used to evaluate the heterogeneity of the study: *I* ^*2*^ < 50%, 50.0–75.0%, and >75.0% represented as low, moderate, and substantial heterogeneity, respectively (Cumpston et al., 2019). Subgroup analysis was conducted based on different types of oral anticoagulants (DOACs or VKAs), and an interaction analysis was applied to evaluate the risk difference between DOACs and VKAs. Sensitivity analyses were performed to strengthen the robustness of the results with the leave-one-out method. Random-effects meta-regression was conducted to test the pre-specified demographic characteristics in order to explore the potential confounding factors associated with all-cause mortality, using a restricted maximum likelihood. The potential publication bias was assessed by evaluating the study effects using visual inspection of funnel plots and Egger’s regression test. The meta-analyses were performed with STATA 15.1 (Stata, College Station, TX, United States).

## 3 Results

### 3.1 Study Selection and Characteristics

**FIGURE 1:**
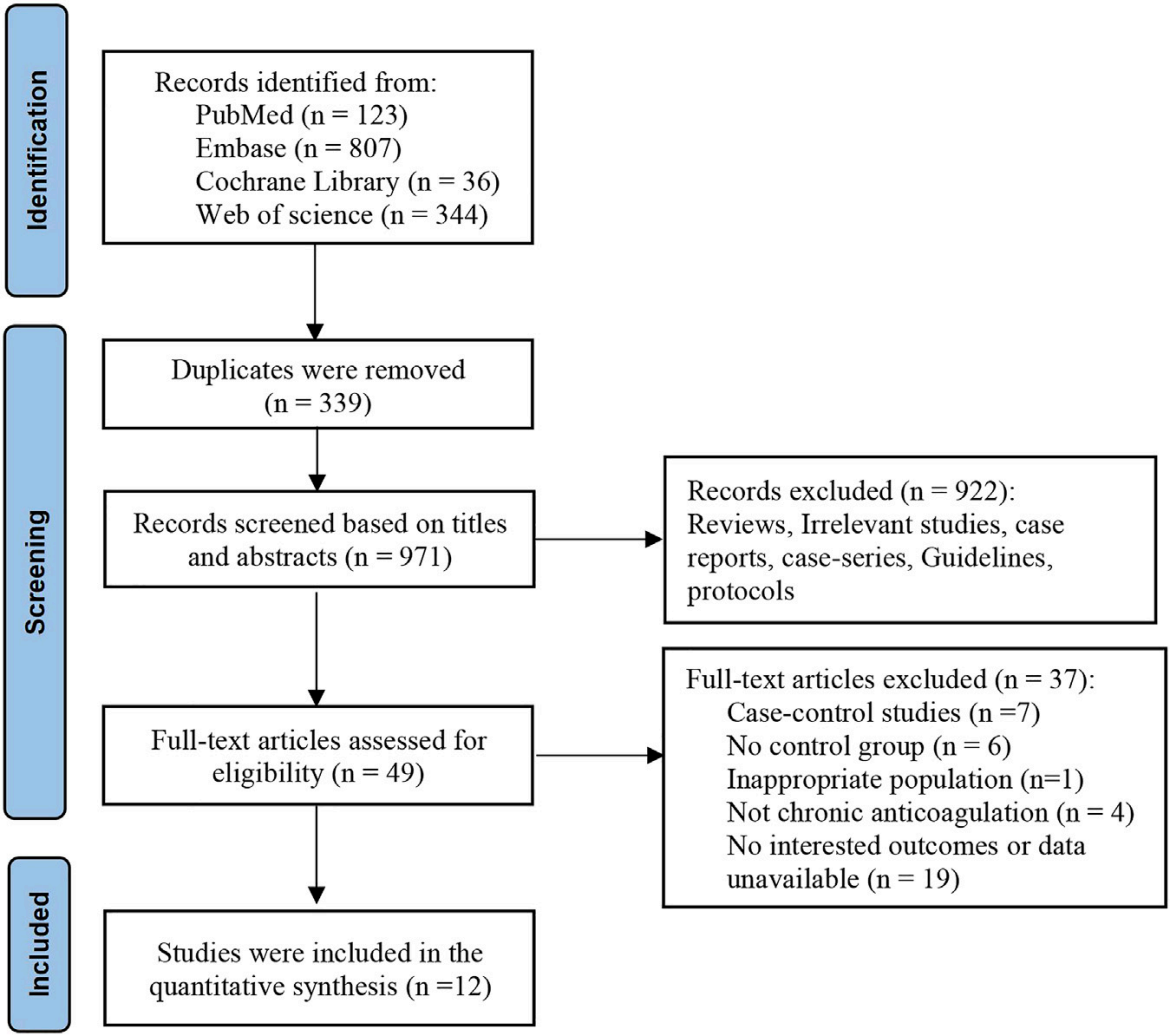
Flow diagram for the selection of studies for the meta-analysis.

A total of 1,310 studies were retrieved from electronic databases through a comprehensive search. After screening the titles and abstracts, 339 duplicates were removed, and 922 records were excluded. After assessing full text, 12 studies (Aslan et al., 2021; Buenen et al., 2021; Chocron et al., 2021; Covino et al., 2021; Denas et al., 2021; Fröhlich et al., 2021; Gülcü et al., 2021; Harrison et al., 2021; Iaccarino et al., 2021; Rivera-Caravaca et al., 2021; Russo et al., 2021; Spiegelenberg et al., 2021) involving 30,646 patients were selected according to the inclusion criteria. The PRISMA flowchart illustrates the results of the literature search and study selection (Figure 1). The characteristics of the included studies are summarized in Table 1. All the 12 included studies were retrospective cohort studies, of which 7 used the propensity score matching (PSM) method to minimize the influence of confounding factors between comparison groups and provided accurate results, and five studies applied the covariate adjustment. Among these, one study was conducted in the United States, while 11 studies were conducted in Europe, including Italy, Turkey, Germany, the Netherlands, and France. The patient demographics and clinical characteristics of included studies are summarized in Table 2. The mean age range of involved patients was 62–84 years, and the proportion of males was 45.6–63.8%. The percentage of comorbidities, such as hypertension, diabetes mellitus, congestive heart failure, pulmonary disease, and renal disease, was reported in the majority of studies. The quality of cohort studies is assessed and summarized in Table 1; all included studies were rated fair and good.

**TABLE 1:** Characteristics of included trials.

Author	Study design	Country	Sample size	Study setting	Type of anticoagulant (%)	Adjusted method	Reported outcomes	Quality
Aslan et al. (2021)	Retrospective cohort	Turkey	1710	Hospitalized	DOACs (100%)	CA	All-cause mortality	8
Buenen et al. (2021)	Retrospective cohort	The Netherlands	497	Emergency department	DOACs (51.8%) and VKAs (48.2%)	CA	All-cause mortality	8
Chocron et al. (2021)	Retrospective cohort	France	2,878	Hospitalized	DOACs (60.7%) and VKAs (39.3%)	PSM	All-cause mortality	7
Covino et al. (2021)	Retrospective cohort	Italy	2,666	Emergency department	DOACs (77.8%) and VKAs (22.2%)	PSM	All-cause mortality	8
Denas et al. (2021)	Retrospective cohort	Italy	4,697	Registration systems	DOACs (21.2%) and VKAs (78.8%)	PSM	ICU admission and all-cause mortality	6
Fröhlich et al. (2021)	Retrospective cohort	Germany	6,637	Registration systems	DOACs (69.5%) and VKAs (30.5%)	CA	All-cause mortality or need for non-invasive or invasive ventilation or ECMO	6
Gülcü et al. (2021)	Retrospective cohort	Turkey	5,575	Hospitalized	DOACs (84.9%) and VKAs (15.1%)	PSM	All-cause mortality	8
Harrison et al. (2021)	Retrospective cohort	America	1,026	Hospitalized	DOACs (78.8) or VKA (21.2%)	CA	All-cause mortality	7
Iaccarino et al. (2021)	Retrospective cohort	Italy	2,377	Registration systems	OAC	CA	All-cause mortality and ICU admission	6
Rivera-Caravaca et al. (2021)	Retrospective cohort	Ecuador, Germany, Italy, and Spain	1,002	Registration systems	DOACs (25.5%) and VKAs (74.5%)	PSM	All-cause mortality and several comorbidities	6
Russo et al. (2021)	Retrospective cohort	Italy	427	Hospitalized	DOACs (62.1%) and VKAs (37.9%)	PSM	All-cause mortality	8
Spiegelenberg et al. (2021)	Retrospective cohort	The Netherlands	1,154	Hospitalized	DOACs (51.6%) and VKAs (48.4%)	PSM	All-cause mortality and ICU admission	6

DOACs, direct oral anticoagulants; OAC, oral anticoagulant; VKAs, vitamin K antagonists; PSM, propensity score matching; CA, covariate adjustment; ICU, intensive care unit; ICH, intracranial hemorrhage; ECMO, extracorporeal membrane oxygenation.

**TABLE 2:** Patient demographics and clinical characteristics of included studies.

Author	Mean age (years)	Male (%)	Hypertension (%)	Diabetes mellitus (%)	Pulmonary disease (%)	Congestive heart failure (%)	Renal disease (%)
Aslan et al. (2021)	62.0	49.5	42.0	27.0	6.0	3.0	NA
Buenen et al. (2021)	71.8	63.8	52.1	20.5	26.0	NA	19.5
Chocron et al. (2021)	66.6	57.9	50.5	23.5	NA	NA	NA
Covino et al. (2021)	84.0	50.0	41.8	18.5	17.4	23.4	15.2
Denas et al. (2021)	NA	50.6	64.1	19.9	NA	6.4	4.8
Gülcü et al. (2021)	64.4	50.2	49.5	26.9	13.9	5.3	2.7
Iaccarino et al. (2021)	68.2	62.7	59.0	18.0	8.0	12.0	6.0
Rivera-Caravaca et al. (2021)	82.0	59.2	82.1	31.2	18.3	4.6	15.1
Russo et al. (2021)	67.0	63.0	62.0	26.0	19.0	8.0	13.0
Spiegelenberg et al. (2021)	69.3	63.7	39.3	22.7	24.1	4.9	NA
Fröhlich et al. (2021)	66.4	50.9	58.7	22.4	11.4	12.8	16.2
Fröhlich et al. (2021)	65.6	51.3	57.0	21.9	11.0	11.9	16.1
Harrison et al. (2021)	76.3	47.5	73.8	38.3	NA	23.4	22.9
Harrison et al. (2021)	76.3	45.6	73.1	38.0	NA	20.8	21.7

NA, not available.

### 3.2 Association of Chronic OAC With All-Cause Mortality in COVID-19 Patients

**FIGURE 2:**
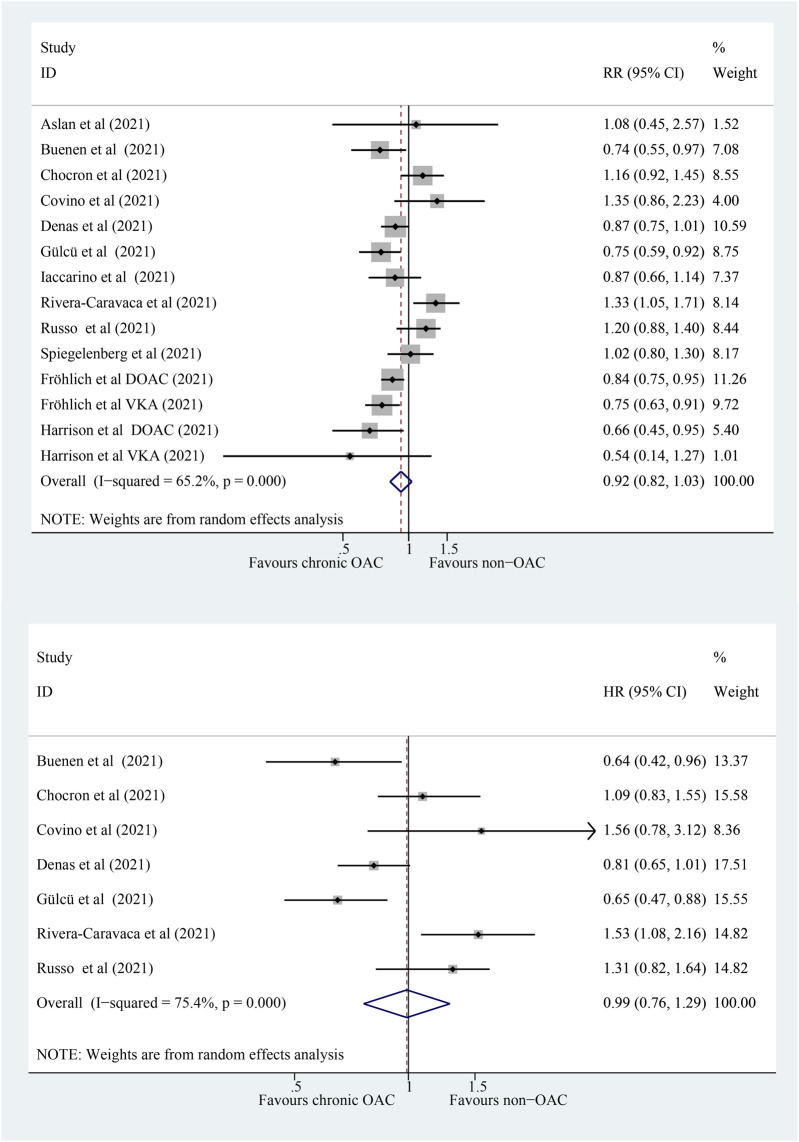
Forest plot for association of chronic OAC with all-cause mortality in COVID-19 patients, random model. **(A)** Pooled the RR. **(B)** Pooled the adjusted HR. OAC, oral anticoagulation; COVID-19, coronavirus disease 2019; RR, relative risk; HR, hazard ratio; CI, confidence interval; DOAC, direct oral anticoagulant; VKA, vitamin K antagonist; *P*, heterogeneity test.

The outcome of all-cause mortality was available in 12 studies (14 cohorts) that compared chronic OAC patients with non-OAC patients with COVID-19. The meta-analysis of studies revealed that patients with chronic OAC were not associated with a significantly lower risk of all-cause mortality than those without OAC (Figure 2A; RR: 0.92; 95% CI: 0.82–1.03; *p* = 0.165; *I* ^*2*^ = 65.2%). A total of seven studies provided adjusted measure as HR, and the pooled HR (Figure 2B; HR: 0.99, 95% CI: 0.76–1.29; *p* = 0.941; *I* ^*2*^ = 75.4%) was consistent with pooled RR. Meta-regression was conducted to test whether demographic characteristics (sample size, mean age, percentage of male, study setting, hypertension, diabetes mellitus, congestive heart failure, pulmonary disease, and renal disease) were associated with all-cause mortality; any potential confounding factors could not be identified (*p* > 0.05 for each characteristic, Supplementary Table S3). The sequential removal of each study failed to identify those with a significant influence on the results (Supplementary Table S4).

### 3.3 Association of Different Types of OACs With All-Cause Mortality

**FIGURE 3:**
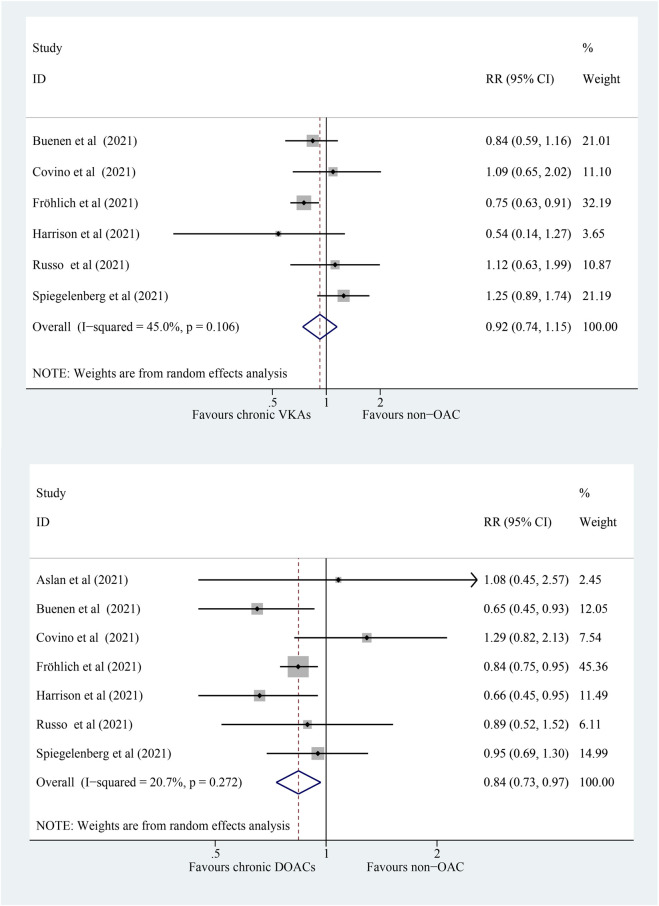
Forest plot for the association of different types of OACs with all-cause mortality in COVID-19 patients, random model. **(A)** Chronic VKAs. **(B)** Chronic DOACs. OAC, oral anticoagulation; COVID-19, coronavirus disease 2019; RR, relative risk; CI, confidence interval; DOACs, direct oral anticoagulants; VKAs, vitamin K antagonists; *P*, heterogeneity test.

The mortality outcome was stratified by DOACs or VKAs. A total of six studies compared the risk of mortality in COVID-19 patients with chronic VKAs with those without OACs. The patients with chronic VKA therapy faced an equal risk of all-cause mortality compared to those without OACs (Figure 3A; RR: 0.92; 95% CI: 0.74–1.15; *p* = 0.463; *I* ^*2*^ = 45.0%). Subsequently, seven studies compared the risk of mortality in chronic DOACs patients with non-OAC patients with COVID-19, and the pooled RR showed a significantly lower all-cause mortality in chronic DOACs than non-OACs in patients with COVID-19 (Figure 3B; RR: 0.84; 95% CI: 0.73–0.97; *p* = 0.015; *I* ^*2*^ = 20.7%). The interaction analysis was conducted to evaluate the risk difference between VKAs and DOAC groups; however, no significant difference was detected (*P*_interaction_ = 0.497).

### 3.4 Association of Chronic OAC With Intensive Care Unit Admission in COVID-19 Patients

**FIGURE 4:**
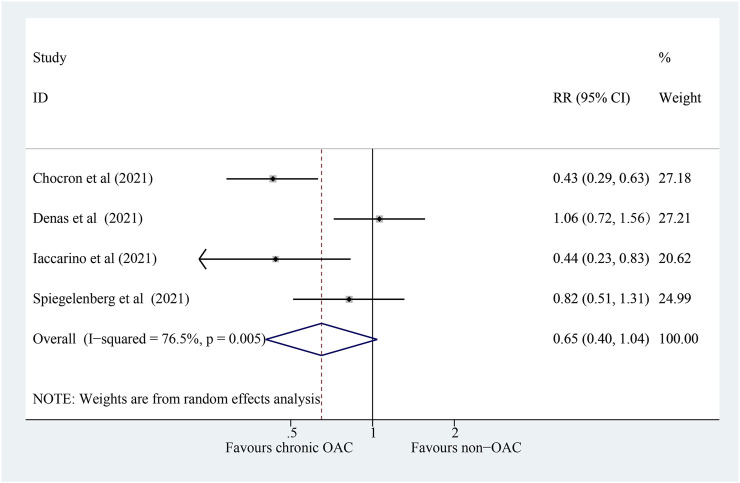
Forest plot for the association of chronic OAC with ICU admission in COVID-19 patients, random model. OAC, oral anticoagulation; COVID-19, coronavirus disease 2019; RR, relative risk; CI, confidence interval; ICU, intensive care unit; *P*, heterogeneity test.

The outcome of ICU admission was available in four studies that compared chronic OAC patients with non-OAC patients with COVID-19. We documented that patients taking chronic OAC could not ameliorate ICU admission significantly (Figure 4; RR: 0.65; 95% CI: 0.40–1.04; *p* = 0.073; *I* ^*2*^ = 76.5%).

### 3.5 Publication Bias

The visual inspection of funnel plots of primary outcomes for publication bias analyses showed a qualitatively symmetrical funnel-plot (Supplementary Figure S1). No significant publication bias was found, as confirmed by Egger’s test (*p* = 0.602).

## 4 Discussion

This study provided a comprehensive investigation of the effectiveness of chronic OAC in COVID-19. The results suggested that chronic OAC could not reduce the high risk of all-cause mortality or ICU admission in patients with COVID-19 compared to those not using OACs. Although the subgroup analysis suggested a potentially reduced risk of mortality in COVID-19 patients with chronic use of DOACs, the interaction analysis did not detect any significant difference between DOACs and VKAs. Together, the results of this study did not support that chronic OAC is beneficial in reducing the risk of mortality in patients with COVID-19.

The high rates of thromboembolic complications were observed in COVID-19 patients. The excess mortality of COVID-19 may be related to the hypercoagulability and microthrombi, leading to lung capillary occlusion (Ackermann et al., 2020; Varga et al., 2020). However, whether patients can benefit from OAC before the early stages of COVID-19 infection remains uncertain. This meta-analysis, based on 12 cohort studies involving 30,646 COVID-19 patients, is the first to provide a comprehensive overview of the effects of chronic OAC. The results demonstrated that chronic OAC does not reduce the all-cause mortality and ICU admission in COVID-19 patients. To the best of our knowledge, only one previous meta-analysis (Kamel et al., 2021) has demonstrated that there was no association between pre-admission anticoagulation and mortality in COVID-19 patients (RR: 0.84, 95% CI: 0.49–1.43; *p* > 0.05), which was consistent with our findings. However, the meta-analysis (Kamel et al., 2021) included only three studies (two were poor quality) with high heterogeneity (*I* ^*2*^ = 86%) and did not elaborate on the effects of the confounding factors that could affect the results, thereby providing weak preliminary conclusions. The evidence for the benefits and risks of an intervention can be obtained from high-quality observational studies when RCTs are lacking (Wei et al., 2018). The present meta-analysis included 12 cohort studies to build a large dataset to provide high-quality evidence, whether chronic OAC could improve the prognosis of patients with COVID-19, and elaborated the influence of the confounding factors by the subgroup analysis and meta-regression to aid in decision-making for the treatment. In the analysis based on drug classification, each subgroup showed acceptable heterogeneity (both *I* ^*2*^ < 50%). The type of OAC might be a source of moderate heterogeneity for the primary outcome (*I* ^*2*^ = 65.2%), but the interaction analysis did not detect any statistical difference between DOACs and VKAs in this study. The meta-regression analysis failed to identify any potential confounding factors on all-cause mortality. However, confounding factors that might not have been reported adequately in the included studies, such as different treatment options for COVID-19, may contribute to the unexplained heterogeneity.

Previous studies proposed that with the direct invasion of SARS-CoV-2 into endothelial cells, the extensive endotheliitis, immune complex accumulation, and type 3 hypersensitivity reaction results in diffuse vascular inflammation (Carsana et al., 2020; Aslan et al., 2021), while the hypercoagulability of COVID-19 is secondary to vascular inflammation. Furthermore, the clotting system activation and the immune-mediated inflammatory response have a complex interplay. The two processes mutually reinforce each other (Scudiero and Parodi, 2020; Hadid et al., 2021) and lead to microvascular thrombosis and death. This might explain why OAC, targeting a single pathway, cannot influence the progression of COVID-19 and eventually the mortality of the infected patients (Aslan et al., 2021; Russo et al., 2021). Thus, rather than against secondary hypercoagulability, directed against thrombogenic inflammation or vasculopathy may be a better treatment option (Flam et al., 2021).

Another study suggested that drugs that inhibit thrombin (coagulation factor IIa) and coagulation factor Xa, such as DOACs, might be promising in ameliorating disease progression and severity of COVID-19. In addition to the prevention of thrombosis, inhibition of factor IIa and factor Xa prevent a cytokine storm (Jose and Manuel, 2020), which might also have an antiviral effect *via* inhibition of SARS-CoV-2 fusion (Frydman et al., 2020). Although VKAs inhibits factors IIa and Xa, vitamin K insufficiency accelerates elastic fiber damage and thrombosis in severe COVID-19 due to impaired activation of matrix Gla protein (protects against pulmonary and vascular elastic fiber damage) and extrahepatic endothelial anticoagulant protein S, respectively, related to poor outcomes (Dofferhoff et al., 2020). However, this meta-analysis is based on real-world studies, suggesting that the impact of these mechanisms on COVID-19 mortality was negligible, and DOACs did not significantly reduce the risk of all-cause mortality compared to VKAs.

Additionally, the chronic anticoagulation indications (mostly by atrial fibrillation and VTE) of patients with COVID-19 were the per se risk factors of mortality (Mountantonakis et al., 2021). The pro-inflammatory effects, a “lethal cocktail,” of COVID-19 render these patients susceptible (Rivera-Caravaca et al., 2021). Patients with anticoagulation indications should be advised to continue OAC during the COVID-19 pandemic, based on the results of this study. The OAC did not increase the mortality due to COVID-19; however, it might be beneficial to patients with anticoagulation indications and reduce the inherent high risk of thromboembolism. In addition, the recommendations from the Italian Federation of Anticoagulation Clinics (Poli et al., 2020) were consistent with those of this study, suggesting to continue OAC for patients with anticoagulation indications in the COVID-19 era. Furthermore, the switch from VKAs to DOACs should be considered in the absence of contraindication because social distancing and stay-at-home orders were implemented. The patients on VKAs were facing unique challenges, who requiring close and continued monitoring by clinic staff to optimize the benefit–risk ratio of warfarin therapy (Kish and Lekic, 2021).

The results need to be interpreted with caution. First, there is a lack of evidence that chronic OAC improves prognosis. Second, the effects of anticoagulants are influenced by genetic factors (Conti et al., 2021), other antithrombotic drugs (Valeria et al., 2021), and potential drug–drug interactions; for example, the drug–drug interactions of anticoagulants with some antiviral treatments used in COVID-19 patients. These phenomena might increase the plasma levels of anticoagulants (Testa et al., 2020), and exposure to these molecules could cause severe bleeding events (Wu et al., 2015). Therefore, OAC during the early stages of COVID-19 infection should not be generalized to all patients in the general population. However, adhering to chronic OAC for patients with thrombotic complications diseases was essential. Several ongoing RCTs are focused on evaluating the potential effectiveness of anticoagulant therapy (including OAC) on the spectrum of COVID-19 patients (Talasaz et al., 2021). This finding needs to be substantiated further by appropriate RCTs.

## 5 Limitations

The present study has several limitations. First, all the included studies were observational studies. Although multiple measures, such as the adjusted effect estimates, subgroup analysis, and meta-regression, were used to reduce the risk of bias of primary analysis. The meta-analysis assembling data from observational studies had some limitations, such as among-study heterogeneity, selection bias, and various confounders. Second, moderate heterogeneity was observed in the primary outcomes, and methodological and clinical heterogeneity could be detected for all meta-analyses, especially involving retrospective studies. Moreover, no highly homogeneous studies support that chronic OAC reduces mortality in patients with COVID-19, and the conclusions of this study were credible. Third, the anticoagulation regimen during hospitalization influences the reported results. However, no standard protocol is yet available for COVID-19 treatment; all medical interventions were performed after hospital admission at the discretion of the medical team, and the results were credible under current treatment conditions. Finally, the assessment of major or non-major clinically relevant bleeding and thromboembolic complications was not performed because data were not available. Therefore, the results were considered with caution since the possibility of confounders could not be excluded.

## 6 Conclusion

The current systematic review and meta-analysis provided evidence that chronic OAC is not associated with improving the prognosis in COVID-19 patients, and the results were consistent across DOACs and VKAs subgroups. RCTs are required to produce high-quality evidence regarding the safety and efficacy of OAC on the full spectrum of COVID-19 patients.

## Data Availability

The original contributions presented in the study are included in the article/Supplementary Material; further inquiries can be directed to the corresponding authors.
