# Supplementary material for: The Use of Oral Anticoagulation Is Not Associated With a Reduced Risk of Mortality in Patients With COVID-19: A Systematic Review and Meta-Analysis of Cohort Studies
Source: Front Pharmacol. 2022 Mar 31;13:781192. doi: 10.3389/fphar.2022.781192 (PMC9008218; doi:10.3389/fphar.2022.781192)
Supplement: Supplementary file 1 [file DataSheet1.docx]

Supplementary Material

**Supplementary Table 1.** PICOS format for the research question

| **P**: Covid -19 patients |
| --- |
| **I**: Oral anticoagulation |
| **C**: No oral anticoagulation |
| **O**: Mortality or ICU admission |
| **S**: RCTs or cohort studies |

**Supplementary Table 2.** Search strategy for the included databases based (PubMed as an example)

| **PubMed (July 24, 2021)** | | |
| --- | --- | --- |
| **Number** | **Searches** | **Results** |
| #3 | #1 AND #2 | 123 |
| #2 | "COVID-19"[MeSH Terms] OR "SARS-CoV-2"[MeSH Terms] OR "2019 novel coronavirus"[Title/Abstract] OR "COVID-19"[Title/Abstract] OR "2019 ncov infection"[Title/Abstract] OR "coronavirus disease 19"[Title/Abstract] OR "2019 novel coronavirus"[Title/Abstract] OR "2019 ncov disease"[Title/Abstract] OR "novel coronavirus"[Title/Abstract] | 151,982 |
| #1 | "Dabigatran"[MeSH Terms] OR "Warfarin"[MeSH Terms] OR "Rivaroxaban"[MeSH Terms] OR "apixaban"[Supplementary Concept] OR "edoxaban"[Supplementary Concept] OR "oral anticoagulants"[Title/Abstract] OR "novel oral anticoagulants"[Title/Abstract] OR "new oral anticoagulants"[Title/Abstract] OR "non vitamin k oral anticoagulants"[Title/Abstract] OR "NOACs"[Title/Abstract] OR "direct oral anticoagulants"[Title/Abstract] OR "DOACs"[Title/Abstract] OR "direct acting oral anticoagulants"[Title/Abstract] OR "vitamin k antagonist"[Title/Abstract] | 35,108 |

**Supplementary Table 3.** Univariable meta-regression for all-cause mortality

| Variables | No. of cohorts | *P* value |
| --- | --- | --- |
| Sample size | 14 | 0.134 |
| Study setting | 14 | 0.782 |
| Mean age | 13 | 0.207 |
| Male | 14 | 0.113 |
| Hypertension | 14 | 0.986 |
| Diabetes mellitus | 14 | 0.772 |
| Congestive heart failure | 12 | 0.354 |
| Pulmonary disease | 10 | 0.476 |
| Renal disease | 11 | 0.832 |

**Supplementary Table 4.** Sensitivity analysis with leave‐one‐out method for all-cause mortality

| Study Omitted | RR (95% CI) |
| --- | --- |
| Aslan et al (2021) | 0.92(0.82-1.03) |
| Buenen et al (2021) | 0.94(0.83-1.06) |
| Chocron et al (2021) | 0.90(0.80-1.01) |
| Covino et al (2021) | 0.91(0.81-1.02) |
| Denas et al (2021) | 0.93(0.82-1.06) |
| Gülcü et al (2021) | 0.94(0.83-1.04) |
| Iaccarino et al (2021) | 0.93(0.82-1.05) |
| Rivera-Caravaca et al b (2021) | 0.90(0.80-1.00) |
| Russo et al (2021) | 0.90(0.80-1.01) |
| Spiegelenberg et al (2021) | 0.91(0.81-1.03) |
| Fröhlich et al DOACs (2021) | 0.93(0.82-1.06) |
| Fröhlich et al VKAs (2021) | 0.94(0.83-1.06) |
| Harrison et al DOACs (2021) | 0.94(0.84-1.06) |
| Harrison et al VKAs (2021) | 0.93(0.83-1.04) |

*RR, relative risk; CI, confidence interval.*


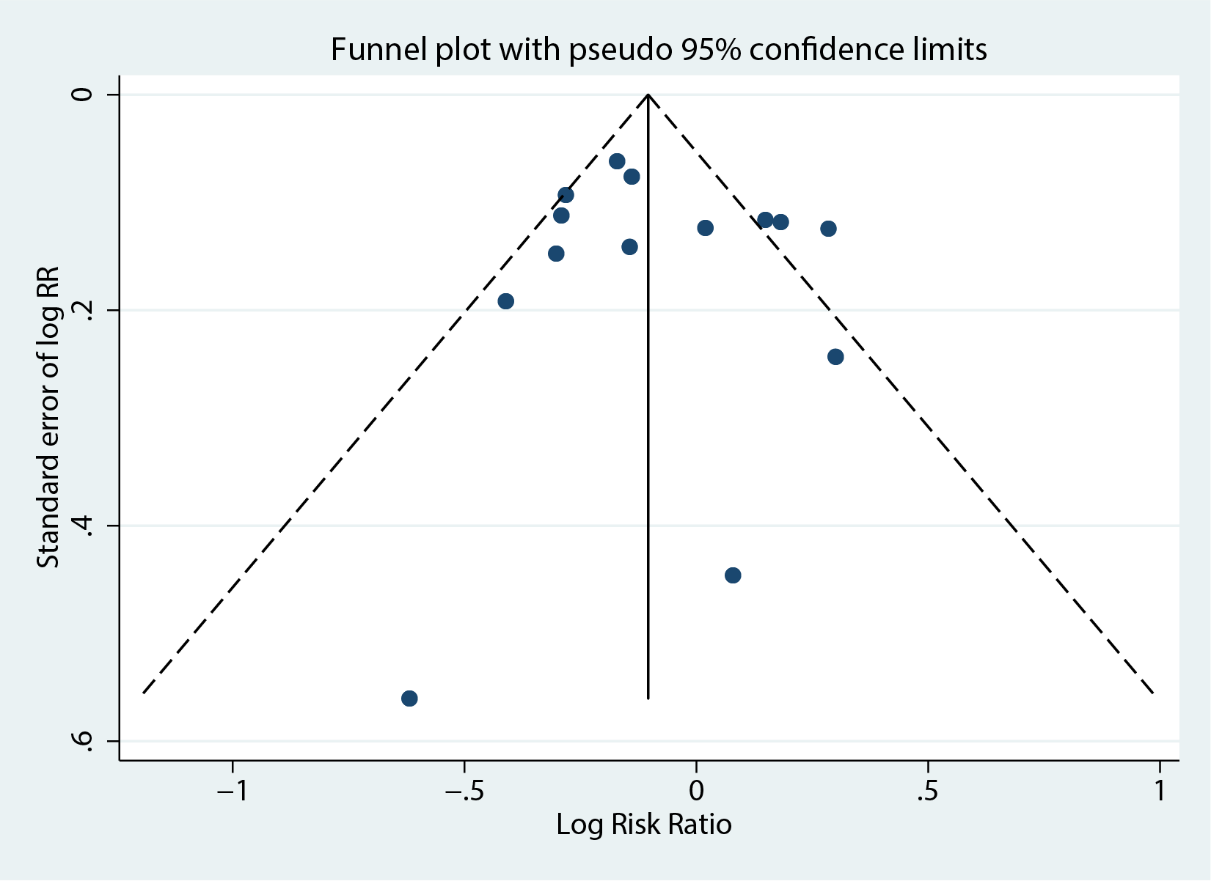


**Supplementary Figure 1.** Funnel plot analysis for the association of chronic OAC with all-cause mortality of COVID-19. OAC oral anticoagulation, COVID-19 coronavirus disease 2019.
